# Influence of Propolis on Hygiene, Gingival Condition, and Oral Microflora in Patients with Cleft Lip and Palate Treated with Fixed Orthodontic Appliances

**DOI:** 10.1155/2013/183915

**Published:** 2013-05-19

**Authors:** Agnieszka Machorowska-Pieniążek, Tadeusz Morawiec, Anna Mertas, Marta Tanasiewicz, Arkadiusz Dziedzic, Wojciech Król

**Affiliations:** ^1^Department of Orthodontics, Medical University of Silesia in Katowice, Plac Traugutta 2, 41-800 Zabrze, Poland; ^2^Department of Oral Surgery, Medical University of Silesia in Katowice, Plac Akademicki 17, 41-902 Bytom, Poland; ^3^Department of Microbiology and Immunology, Medical University of Silesia in Katowice, Jordana 19, 41-808 Zabrze, Poland; ^4^Department of Conservative Dentistry with Endodontics, Medical University of Silesia in Katowice, Plac Akademicki 17, 41-902 Bytom, Poland

## Abstract

The aim of this study was to evaluate the influence of 3% ethanol extract of propolis (EEP) on hygiene, gingival and microbiological status of oral cavity in patients with cleft lip and palate treated with fixed orthodontic appliances. The study included forty-one nonsyndromic complete unilateral of bilateral cleft lip and palate subjects with fixed appliance on at least 10 teeth. Twenty-one subjects were instructed to brush their teeth three times a day using toothpaste with propolis. Control group included twenty subjects who were asked to brush their teeth three times a day using a toothpaste without propolis. API, OPI, GI, and supragingival bacterial plaque were taken from each subject twice: baseline and after using the toothpaste for 35 days. The final examinations showed statistically significant decrease in OPI, GI, and the percentage of the *Actinomyces* spp. and *Capnocytophaga* spp. compared with baseline in propolis group subjects. The improvement in oral health in these patients confirms antibacterial, anti-inflammatory, and regenerative properties of propolis.

## 1. Introduction

The purpose of orthodontic therapy is to obtain a correct occlusion, harmonious facial contours, and efficient stomatognathic system with healthy periodontium and no dental caries. The presence of malocclusion, in particular teeth crowding accompanied by orthodontic appliances, leads to accumulation of dental plaque and problems with self-purification of teeth [1–5]. Orthodontic appliances modify oral environment affecting the amount, flow, and composition of saliva, including its pH and buffer ability, and induce occurrence of blood in saliva [6, 7]. Furthermore, orthodontic therapy changes oral bacterial flora [8–10]. Brackets, bands, bars, wires, and other components of an orthodontic appliances may cause iatrogenic gingival swelling and are responsible for additional dental plaque retentions which are hardly accessible for mechanical cleaning of teeth [11–13]. Changes within oral cavity, occurring as a result of orthodontic treatment, may lead to diseases in dental hard tissues or in periodontium and mucosa. Therefore wearing fixed orthodontic appliances requires careful oral hygiene every day using brushes, irrigators, pastes, and mouthwashes. Pharmaceutical industry continually creates new chemical preparations and compounds to help maintaining proper oral hygiene. However, a great help comes from the nature as well.

Products obtained from plants or animals have been arousing much interest [14]. One of such products is propolis. Its beneficial properties were known and used already in the ancient times [15]. The Greek, Romans, and Egyptians used propolis to cure cuts, nonhealing wounds, or ulcers and to embalm corpses [16]. Propolis is produced by bees from plant buds or cracks in tree barks. It is then modified enzymatically and used to seal up their beehive doors, line beehive walls, or protect against microorganisms [17]. Propolis is a dense, adhesive mixture of wax and resin consisting of plant balsams, volatile oils, and chemically active compounds like phenolic acid or their esters, flavonoids (flavones, flavanones, and flavanols), aromatic alcohols and aldehydes, terpenes, fatty acids, *β*-steroids, mineral salts, and vitamins [15, 18]. Propolis composition is varying and depends upon bee species, plant species, and climate [15, 17]. Propolis has strong bacteriocidal, antiviral, antiparasitic, fungicidal, and antioxidative properties. In vivo and in vitro studies confirmed anti-inflammatory properties of propolis and showed its strong immunomodulating effects [17–20]. Phenolic acids, aldehydes, ketones, and flavonoids inhibit classic and alternative complement activation and stimulate production of antibodies and INF*γ* synthesis [21]. Prenylated p-coumaric acid activates macrophages, and caffeoylquinic acid derivatives stimulate their motility and spreading [22]. Furthermore, ethanol extract of propolis (EEP) was found to have an anti-inflammatory effect through IL-1*β* m RNA expression inhibition and nitric oxide synthase (iONS) together with scavenging free radicals produced by neutrophils and macrophages [23–26]. Its antitumor effects were confirmed by many authors [27–31]. Considering the very wide range of therapeutic properties of propolis, a decision was taken to evaluate its influence on oral condition during orthodontic treatment.

The purpose of this paper is to evaluate the influence of 3% ethanol extract of propolis (EEP) on hygiene, gingival and microbiological status of oral cavity in patients with cleft lip and palate treated with fixed multiband-bracket appliances.

## 2. Materials and Methods

### 2.1. Clinical Examinations

The examinations were performed in Orthodontic Outpatient Clinic, Academic Center of Dentistry, and Specialist Medicine in Bytom, Poland. The study group consisted of 41 patients with nonsyndromic complete unilateral of bilateral cleft lip and palate (CLP) treated with fixed appliances. All patients had a fixed appliance on at least 10 teeth; they were in good general condition and had undergone no antibiotic therapy or surgical treatment of the face for at least one month before. Mean age was 12.37 years, and there were 17 girls and 24 boys (Table 1). The patients were divided randomly into two groups: propolis group (21 patients) and control group (20 patients). Propolis group patients were instructed to use CT gel, Carepolis toothpaste with propolis, and control group patients CC gel, Carepolis toothpaste without propolis. Dental toothpaste with CT propolis had 3% content of ethanol extract of Brazilian propolis. Raw propolis was collected from the beekeeping section of the Seiri Alimentos Naturales, Brazil. Propolis samples were obtained from colonies of Africanized honeybees (*Apis mellifera*) in Minas Gerais State, Southeast Brazil, and collected in 2008 from the plant Baccharis dracunculifolia using plastic net. The unprocessed propolis was sent to the Nihon Natural Therapy Co., Ltd., Tokyo, Japan, for preparation of the EEP. The toothpastes with 3% of EEP and without of EEP (placebo) were prepared in Nippon Zettoc Co., Ltd., Tokyo, Japan.

At baseline each patient received general instructions on the oral hygiene and was told to clean the teeth using Fones method, to use interdental brushes, and to clean the teeth three times daily using the toothpaste. The patients were informed about the purpose and method of the study and agreed to participate. The research programme was approved by the Bioethics Committee of the Silesian Chamber of Medicine (resolution no. 6/2010).

Oral hygiene and gingivae were evaluated, and a sample for microbiological examination was taken from each patient in both groups twice: at the baseline and after 35 days of using the paste. The examinations were performed by one investigator, with the same lighting, using a mirror, probe, and bead probe. Oral hygiene was examined using modified (without staining) Approximate Plaque Index (API) according to Lange and Orthodontic Plaque Index (OPI) for the segment of incisors and canines [32, 33]. 

Additionally, marginal gingivae was examined in each patient using gingival index (GI) according to Löe and Silness [34]. Furthermore, supragingival bacterial plaque was taken using a disposable microbiological swab set. Deposit was taken, using a sterile cotton swab rod, from gingival margin of the buccal surfaces of teeth 14 or 15 where orthodontic brackets were placed.

### 2.2. Microbiological Examinations

Samples for microbiological testing were inoculated on suitable culture media (Columbia agar, Schaedler K3 agar, and Sabauraud agar) from Biomerieux (Marcy l'Etoile, France). Aerobic bacteria were propagated on Columbia agar medium with 5% sheep blood at 37°C. Anaerobic bacteria were propagated on Schaedler K3 medium with 5% sheep blood at 37°C in anaerobic conditions using Genbaganaer (Biomerieux, Marcy l'Etoile, France). *Candida* fungi were propagated on selective Sabouraud agar medium at 35°C in aerobic condition. Upon isolation and further culture of each microorganism, their species were identified with the help of the following reagent sets: Api 20 E, Api 20 NE, Api *Candida* (Biomerieux, Marcy l'Etoile, France), and ENTERO test 24 N, NEFERM test 24 N, STREPTO test 24, and ANAERO test 23 (Erba-Lachema, Brno, Czech Republic).

### 2.3. Data Analysis

Mean values ± standard deviation of minimum and maximum values of each index were measured. W. Shapiro-Wilk test was used to assess distribution normality of the variables. Analysis of the variables within the groups was done using *t*-test for dependent samples in case of normal distribution variables and Wilcoxon matched pairs test in absence of normal distribution. No homogeneous variance was shown (Levene's test: *P* < .01) for all variables. Comparison of the variables between propolis group and control group was performed using Tukey test for normal distribution variables and Mann-Whitney *U* Test for nonparametric variables. All tests were significant with *P* < .05. Statistical analysis was done using Statistica v.8 software (Silesian Medical University, Katowice, Poland).

## 3. Results

### 3.1. Baseline

**Figure 1:**
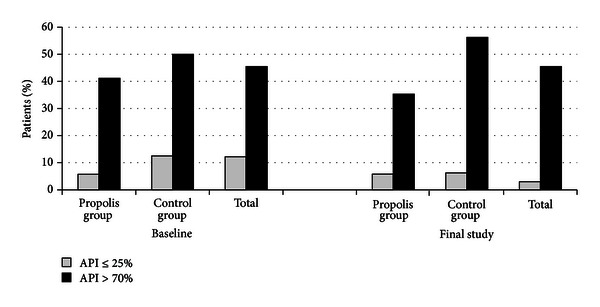
Percentage of patients distributed according to severity of API.

**Figure 2:**
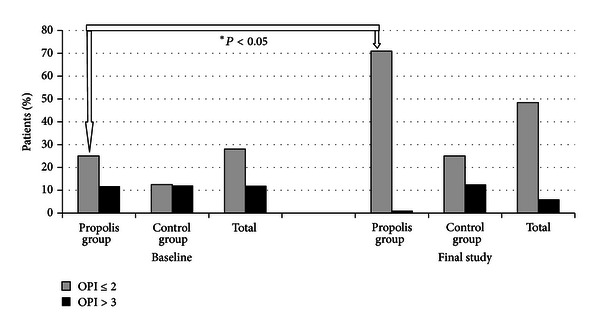
Percentage of patients distributed according to severity of OPI.

**Figure 3:**
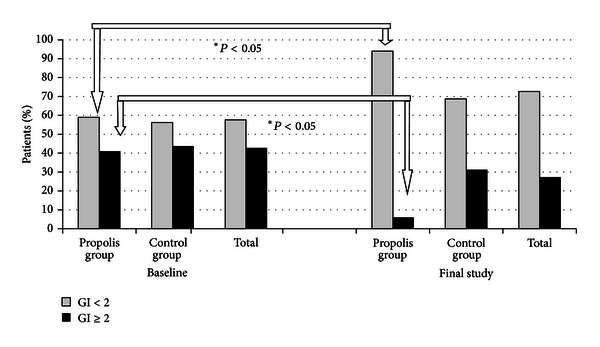
Percentage of patients distributed according to severity GI.

The examinations showed that mean values of all indices, that is, API, GI, and OPI, did not differ statistically between propolis group and control group at the first stage (Tables 2 and 3). Poor oral hygiene (API > 70%) was found in 45.5% of the patients and very good hygiene (API ≤ 25%) in 12.2% of the patients (Figure 1). Low amount of deposit around orthodontic brackets (OPI ≤ 2) was shown in 28% of the patients, while high amount of deposit (OPI > 3) in 11.9% (Figure 2). Low gingival index, confirming good condition of gingivae (GI < 2), was shown in 58% of the patients and high gingival index (GI ≥ 2) in 43% ( Figure 3).

### 3.2. Final Study

Second-stage examinations performed after 35 days showed differences between propolis group and control group. GI and OPI indices were statistically lower in propolis group as compared with control group (*P* < .05). API showed no statistically significant difference between the groups.

### 3.3. Assessment of Propolis Influence on Dental Plaque and Gingivae

Using the paste without propolis (control group) or with propolis (propolis group) had no statistically significant influence on API (*P* > .05) (Table 3). However, statistically significant decreases were detected in propolis patients with reference to OPI and GI (*P* < .05) (Table 3). Furthermore the changes in API, OPI, and GI between the baseline and the final study in both groups were calculated, and a comparison between the propolis group and the control group group was made. API changes did not show a statistically significant difference between the groups (Table 3). However, GI and OPI changes were statistically different between propolis group and control group (*P* < .05). Afterwards, the patients with extreme mean values of API, OPI, and GI were compared. The number of patients with very good oral hygiene and healthy gingivae (API ≤ 25%, OPI ≤ 2, and GI < 2) and with poor hygiene and gingival inflammation (API > 70%, OPI > 3, and GI ≥ 2) was compared in propolis group and control group at the baseline and final stage (Figures 1–3). The greatest changes were in the propolis group. The percentage of patients with very good oral hygiene (OPI < 2) and gingivae without bleeding (GI < 2) was higher and the percentage of patients with gingival inflammation (GI ≥ 2) was lower after using the toothpaste with propolis for 35 days (Figures 2 and 3).

### 3.4. Microbiological Findings

The bacteria most often found in oral swabs in both groups and at both stages of the study were *Streptococcus* spp. and *Neisseria* spp. (Table 4). No cariogenic *Streptococcus mutans* or *Lactobacillus acidophilus* were found in the samples.

Among bacteria particularly pathogenic for parodontal tissues, the presence of *Actinomyces* spp. together with *Actinomyces israelii*, *Capnocytophaga* spp., *Fusobacterium, Bacteroides*, and *Eubacterium* was predominant. Microbiological status was similar in both groups at the first stage of the study. However, the patients using propolis paste had statistically lower levels of *Actinomyces* spp. with *Actinomyces israelii* and *Capnocytophaga* spp. at the second stage of the study. A 10% decrease in *Actinomyces* israelii level accompanied by an increase in *Actinomyces* spp. was found among control patients. The number of *Candida albicans* did not change (Table 4).

## 4. Discussion

Cleft lip and palate (CLP) is a fairly common congenital malformation within the head and neck, the frequency being 1.0–2.21 in 1000 live births [35]. Orthodontic care begins shortly after birth, taking many years and involving a variety of specialists in medicine and oral medicine [36]. Evaluation of oral hygiene and periodontal health in children and adults is an interesting subject for investigators and clinicians [36–48]. The authors of this paper decided to evaluate the influence of tooth cleaning with propolis paste on oral hygiene and oral microflora in patients with CLP.

The baseline examinations showed that most patients in propolis group and control group had poor oral hygiene (API > 70%) with mean API of 64.31%. The mean value of API was slightly higher in cleft palate/cleft lip palate patients studied by Schultes et al. with a tendency to poor oral hygiene [37]. On the other hand, very good oral hygiene (API) was shown in 12.5% of our patients at the baseline study. Slightly higher percentage of optimal oral hygiene was found by Stec et al. among oral cleft children in Łódź in active phase orthodontic treatment with the limit value for good hygiene (API) a little over 40%, so higher than the percentage taken in this paper [38]. Low percentage of children with good oral hygiene and high mean plaque index, detected by our studies and by other authors as well, may be connected with different oral morphology and function in children with oral cleft as compared to children without any cleft. Oral deformity, manifested by nasal communications, frequent surgical procedures, scars within lips and palate, malocclusions, and disturbances in structure, morphology, number, or position of teeth, often accompanied by very long orthodontic treatment, makes oral hygiene maintaining extremely difficult [36]. Components of fixed appliances like bands or other enamel bonded attachments like brackets are also a problem to oral hygiene [11, 12]. Klukowska et al. reported 2-3 times higher level of plaque in patients wearing orthodontic appliances as compared to patients without treatment [39].

OPI used in our study is particularly recommended to assess the level of plaque in patients wearing fixed appliances because its value depends on the presence of dental deposits on each tooth surface adjacent to the bracket base [33]. We used a modified OPI only for anterior segment, and we demonstrated that the baseline value of this index was 2.1 in both groups, indicating a moderately good oral hygiene. No reports on this index, with reference to patients with oral clefts, have been found in the available literature so far.

Gingivae condition was examined using GI, and its baseline mean value was 1.71. Slightly higher value of this index was reported by Costa et al. for children with oral clefts [40]. Their value was statistically higher compared to children without clefts. Our examinations showed that 58% of the patients in both groups had good gingival state, whereas 43% had gingival inflammation. Similar results were received by Perdikogianni et al. and showed that children with cleft lip and palate had moderate gingival inflammation, with no statistical difference compared to children with no cleft [41].

Final-stage examinations showed that mean value of API was slightly lower in both groups. OPI and GI values were statistically lower in propolis group, after using propolis toothpaste for 35 days, as compared to the baseline values. Similar results were received by Dodwad and Kukreja in their study on five-day use of propolis mouth wash [42]. Tanasiewicz et al. demonstrated a beneficial effect of propolis in patients with healthy periodontium and those with periodontitis [43]. Beneficial influence of propolis on periodontium was also demonstrated by Botushanov et al. who studied 42 patients cleaning their teeth with silicate toothpaste with extract from propolis for 28 days [44]. Pereira et al. assessed gingival index and plaque index in patients who had been using alcohol-free mouthwash containing 5.0% Brazilian green propolis for 45 and 90 days. On day 45 and on day 90 the gingival index and plaque index had statistically higher values compared to the baseline [45].

The influence of propolis on patients showing extreme GI values was also studied in both groups. In this study statistical reduction of percentage of patients using propolis tooth-paste were found to have gingival inflammation. Similar situation was noted in control group, but decrease in gingival inflammation cases was not statistically significant. Another interesting observation was that gingivae condition became much better with no significant decrease in dental plaque amount expressed by API. Similar changes were reported by Stec et al. who demonstrated an absence of relationship between API and gingivae condition [38]. It is important to realize that API only gives information about the presence or absence of dental plaque with no assessment of its amount and location.

In final stage of the study OPI showed statistically lower levels than those in the baseline in patients using propolis toothpaste. Other authors also demonstrated a beneficial influence of propolis on the plaque index (PI) [42–44].

Bacterial flora harvested during first-stage examinations in both groups consisted largely of Gram-negative cocci, chiefly *Neisseria* spp., and Gram-positive facultative anaerobes, including chiefly *Streptococcus* spp. and *Actinomyces* spp. Similar results were received by Ritz who demonstrated variable composition of the plaque in accordance with its maturity and predominance of *Neisseria* in the earliest stage of bacterial flora formation [46]. Coexistence of *Streptococcus* spp. and *Actinomyces* spp. is widely reported in the available literature. Perdikogianni et al. detected high mutual proportion of *Streptococcus* and *Actinomyces* in their study on frequency of bacteria in patients with cleft lip and/or palate [41]. Aas et al. demonstrated coexistence of both species in the early phase of dental caries, being to a great extent responsible for originating the process [47]. Cisar et al. revealed the adhesion-receptor mechanism of both bacteria which populate the oral environment through coaggregation [48]. Absence of *Streptococcus mutans* and *Lactobacillus acidophilus* with predominance of *Streptococcus* spp. and *Actinomyces* spp. in our study material confirms the “ecological hypothesis of dental plaque” suggesting that plaque status is a result of an interaction between many different bacterial species currently existing in oral cavity whereas its composition is variable and depends on environmental factors [49]. Munson et al. detected, based on their examinations, the permanent presence of only one or two *Lactobacillus* spp. in each lesion in a subject, and Aas et al. found *Streptococcus mutans* in only 10–20% of their patients with severe caries [47, 50]. The microbiological cultures detected absence of *A. actinomycetemcomitans* in CLP children of this study, but multiform Gram-negative anaerobic bacteria with many pathogens were likely to cause periodontitis. This result was in agreement with the studies on microflora in subjects with oral clefts reported by other investigators [40, 41]. Also, the investigators demonstrated that the composition of oral microflora was individually depending on the sample site, current health state, or tooth condition [9, 11, 47–50]. Ristic et al. indicated lower level of bacterial flora in the region of molars in patients wearing fixed orthodontic appliances, and Alexander detected statistically more cases of gingivitis around molars with orthodontic bands that were around enamel bonded attachments [9, 11].

Final microbiological analysis of bacterial flora in patients using propolis toothpaste revealed the greatest decrease in frequency of Gram-positive facultative anaerobic cocci and Gram-positive facultative anaerobic rods. This result may indicate antibacterial properties of propolis used for every-day oral hygiene and confirmed by in vitro examinations [51–54]. The mechanism of antibacterial effects of propolis is complex and not quite clear [51]. Mirzoeva et al. have demon-strated that the effects of propolis are species-dependent and strongly related to disintegration of bacterial cell membrane through an increase in its permeability by ions. As a result a bacterial cell may lose its membrane potential thus also losing motility and virulence. Such effects are found with flavonoid and cinnamic components of propolis [52]. Other investigators suggest that antibacterial properties of propolis may be related to some additional mechanisms like inhibition of glucosyltransferase synthesis and production of polysaccharide by *Streptococcus* [52–54]. It is important to note that a decrease in the number of bacteria is accompanied by lower severity of gingivitis in patients using propolis. No significant changes in the number of bacteria and no improvement in the condition of gingivae or hygiene were noted among patients using toothpaste without propolis.

## 5. Conclusion

Results of the study examinations and tests reveal a significant improvement in gingivae, a lower level of dental plaque, and a decrease in frequency of Gram-positive rods in children with cleft lip and palate after using propolis toothpaste. The improvement in oral health in these patients confirms antibacterial, anti-inflammatory, and regenerative properties of propolis.

## Figures and Tables

**Table 1:** Demographic data.

	Age				Gender		Total
	Mean	Minimum	Maximum	Std. dev.	Girls	Boys	
Propolis group	12.43	9.8	16.4	1.60	8 19%	13 32%	21 51%
Control group	12.53	9.7	18.2	2.75	9 22%	11 27%	20 49%

Total	12.37	9.7	18.2	2.28	17 41%	24 59%	41 100%

**Table 2:** Summary scores of API, OPI, and GI in baseline and final study.

	Baseline			Final study		
	API%	OPI	GI	API%	OPI	GI
Propolis group *N* = 21						
Mean	64.14	2.14	1.74	62.00	1.69	1.14
Minimum	16.6	0.33	0.16	10.6	1.25	0.16
Maximum	10.0	4.00	2.66	100.0	2.80	2.83
Std.dev.	29.98	0.65	0.51	28.43	0.43	0.48
Control group *N* = 20						
Mean	64.48	2.25	1.69	63.94	2.21	1.61
Minimum	13.9	0.16	0.16	8.33	0.16	0.16
Maximum	100.0	3.16	2.83	100.0	4.00	2.83
Std.dev.	28.01	0.61	0.54	24.46	0.71	0.68
Mean ± std.dev. *N* = 41	64.31 ± 28.73	2.19 ± 0.68	1.71 ± 0.52	63.97 ± 26.44	1.89 ± 0.58	1.37 ± 0.58

**Table 3:** Statistical comparisons of API, OPI, and GI.

		A	B		C
		Intergroup difference	Intragroup difference		Difference test
			Propolis group	Control group	Effect of propolis
API	Baseline final study	0.503^a^ 0.850^a^	0.871^c^	0.871^c^	0.950^b^
		
OPI	Baseline final study	0.137^b^ 0.020^∗,b^	0.012^∗,d^	0.410^d^	0.001^∗,b^
		
GI	Baseline final study	0.063^a^ 0.034^∗, a^	0.014^∗,c^	0.722^c^	0.002^∗,b^
		

A: differences between the propolis group and the control group; B: differences between baseline and final study; C: statistical changes between baseline and final study in the propolis group compared with the control group. ^a^Tukey test, ^b^Mann-Whitney *U*  Test, ^c^ *t*-test for dependent samples, and ^d^Wilcoxon matched pairs test; *significance (*P* < .05).

**Table 4:** Frequency of bacterial species in propolis group and control group in baseline and final study.

Bacterial species	Propolis group *N* = 21		Control group *N* = 20	
	Baseline	Final study	Baseline	Final study
	%	%	%	%
Gram+				
Facultative anaerobic				
*Streptococcus* spp.	90.4	85.7	90.0	100.0
* S. mitis *	57.10	61.9	55.0	60.0
* S. salivarius *	19.04	9.52	25.0	20.0
* S. vestibularis *	19.04	9.52	20.0	30.0
* S. oralis *	19.04	9.52	15.0	10.0
* S. sanguinis *	0.00	9.52	10.0	20.0
*Actinomyces* spp.	28.57	9.52*	30.0	40.0
*Actinomyces israelii *	19.00	0.00*	15.0	25.0
Anaerobic				
*Bifidobacterium* spp.	23.80	23.80	30.0	25.0
* Eubacterium* spp.	4.76	0.00	10.0	10.0
* Gemella morbillorum *	9.52	0.00	15.0	15.0
* Clostridium* spp.	14.28	9.52	0.00	0.00
Gram−				
Facultative anaerobic				
*Neisseria* spp.	90.4	95.2	95.0	90.0
*Capnocytophaga *spp.	19.04	0.00*	15.0	10.0
* Enterobacter kobei *	0.00	0.00	5.0	5.0
* Klebsiella pneumoniae *	0.00	0.00	10.0	10.0
* Klebsiella oxytoca *	4.76	9.52	5.0	10.0
Anaerobic				
*Veillonella* spp.	23.80	23.80	20.0	25.0
*Mitsuokella* spp.	9.52	4.76	0.00	0.00
*Bacteroides* spp.	9.52	9.52	0.0	10.0
*Fusobacterium* spp.	14.28	0.00	25.0	30.0
*Candida *				
*Candida albicans *	9.52	9.52	15.0	15.0

*Significance *P* < .05 compared with baseline.
